# Prevalence of Iron Deficiency and Iron Deficiency Anemia Among Nursing Students Working in the Internal Medicine Clinic

**DOI:** 10.7759/cureus.51212

**Published:** 2023-12-28

**Authors:** Betül Cigdem Yortanlı, Samil Ecirli, Zekiye Soykan Sert

**Affiliations:** 1 Department of Internal Medicine, Konya City Hospital, Konya, TUR; 2 Department of Internal Medicine, Private Academy Meram Hospital, Konya, TUR; 3 Department of Gynecology and Obstetrics, Aksaray University School of Medicine, Aksaray, TUR

**Keywords:** nursing student, deficiency, iron, internal medicine, anemia

## Abstract

Objective

The objective of this study is to evaluate the frequency of iron deficiency (ID) and iron deficiency anemia (IDA) among nursing students working in the internal medicine clinic of our hospital.

Methods

The study was designed as a prospective cohort study. The study population comprised nursing students working in the internal medicine clinic of our hospital from January 1, 2016, through June 1, 2016. Data concerning the participants’ anamnesis, physical examination findings, and sociodemographic characteristics were recorded. Blood samples were taken. The participants' blood parameters were evaluated for anemia.

Results

A total of 99 nursing students, 43 (43.4%) male and 56 (56.6%) female, were included in the study. The mean age was 17.4 ± 0.58 years for the male student group and 17.64 ± 0.73 years for the female student group. We detected ID in 22.2% of the nursing students, anemia in 8%, and IDA in 4%. ID was present in 22 (39.3%) of the female students. There was a significant difference between the male and female student groups in terms of the prevalence of ID (p < 0.001). The mean hemoglobin levels of the male and female student groups were 15.85 ± 1.10 g/dL and 12.90 ± 1.05 g/dL, respectively.

Conclusion

We determined that the number of students with ID was higher than the number of students with IDA. Once ID is diagnosed, it may be necessary to take precautions and treat it according to the severity of the deficiency. It is extremely important to educate nursing students and raise their awareness about ID and IDA.

## Introduction

Anemia is a major public health problem across the world, primarily caused by iron deficiency anemia (IDA). The prevalence of IDA in a country is related to the cultural, socioeconomic, and developmental characteristics of that country [1,2]. The prevalence of IDA varies depending on age, gender, geographical region, cultural habits, and socioeconomic status [1,2]. Studies on IDA have generally been conducted to determine the prevalence of this condition in certain age groups (e.g., newborns, infants, primary school children, pregnant women, and women of specific age ranges). These studies have generally investigated the etiology and frequency of IDA [3-6].

Common symptoms of IDA include fatigue, exercise-related shortness of breath, cold intolerance, loss of appetite, drowsiness, pale skin, headache, tinnitus, impairments in cognitive and intellectual functions, drowsiness, apathy, irritability, and difficulty focusing [7]. Fatigue caused by tissue hypoxia due to anemia, if not controlled, negatively affects the individual’s daily living activities and quality of life. Therefore, it is important to identify, treat, and prevent IDA early among nurses who administer the treatment prescribed by physicians to patients. Considering the negative effects of IDA on physical activity, work performance, and the workforce, this study aimed to evaluate the prevalence of IDA, among nursing students working in our hospital to determine the factors affecting anemia and take early measures for treatment.

## Materials and methods

Study design and participants

This prospective study was conducted in the internal medicine clinic of our hospital from January 1, 2016, to June 1, 2016. The study population consisted of nursing students actively working in our clinic. One nursing student was excluded from the sample since she decided to withdraw from the study. Sample selection was made using the simple random method in proportion to the number of nursing students in the clinics, and 99 nursing students were included in the study. Ethical committee approval was obtained from the Meram University Medical Faculty, Hospital Ethics Committee with the decision number 2016/04-09. Individuals with a known chronic disease or a diagnosis of IDA, those who had previously received IDA treatment, those with IDA or iron deficiency (ID) due to heavy or prolonged uterine bleeding, those who withdrew their consent, and those who did not want to participate in the study were excluded. The participants were provided with written and verbal information about the study, and their written consent was obtained.

Data collection and process

The detailed clinical history and physical examination findings of the participants were obtained. Data concerning sociodemographic characteristics (age, height, weight, nutritional status, daily tea consumption, coffee consumption, smoking, the presence of pica, medications used, and the presence of parasites) were recorded. The female nurses were additionally questioned about the age of first menstruation, menstrual cycle, the presence of juvenile or breakthrough bleeding, any contraceptive use, pregnancy history, and breastfeeding status.

Venous blood samples were taken from nursing students between 8 and 10 a.m. Hemoglobin, mean corpuscular volume (MCV), iron, unsaturated iron-binding capacity (UIBC), vitamin B12, folic acid, and ferritin levels were evaluated in these samples. In addition, peripheral smears were used to examine the erythrocytes, leukocytes, and platelets in blood in terms of their shape, structure, and size. Stool samples were examined for the presence of occult blood and parasites. Throughout the study period, IDA and ID treatment and follow-up of the participants were performed in accordance with standard guidelines.

Definitions

Individuals with a hemoglobin level below 12 g/dL for women and 13 g/dL for men were diagnosed with anemia [2]. According to World Health Organization (WHO) guidelines, healthy students with ferritin levels below 15 µg/L were diagnosed with ID [8]. The coexistence of ID and anemia was defined as IDA. Vitamin B12 deficiency was defined based on its level being below 200 pg/mL, and folic acid deficiency was accepted as a folic acid level below 5.38 ng/mL [9,10]. Transferrin saturation was calculated with the following formula: serum iron / iron-binding capacity x 100.

Data analysis

IBM SPSS Statistics for Windows, Version 22 (Released 2013; IBM Corp., Armonk, New York, United States) was used for statistical analysis. Descriptive statistics were given as mean ± standard deviation for normally distributed data; median (interquartile range) for non-normally distributed data; and number and percentage values for categorical variables. The suitability of the data for a normal distribution was evaluated with the Kolmogorov-Smirnov test. The chi-square test was performed on categorical data. Student’s t-test was used to compare normally distributed data between two groups, and the Mann-Whitney U test was used to compare non-normally distributed data. In comparisons involving more than two independent groups, a one-way analysis of variance was used for variables with a normal distribution and the Kruskal-Wallis test for those without a normal distribution. The statistical significance level was accepted as p < 0.05.

## Results

A total of 99 nursing students, 43 (43.4%) male and 56 (56.6%) female, were included in the study. The mean age was 17.4 ± 0.58 years for the male nursing students and 17.64 ± 0.73 years for the female nursing students. The female and male groups were similar in terms of age, accommodation, coffee consumption, and palpitations. There was a significant difference between the female and male groups in terms of height (p <0.001), weight (p < 0.001), tea consumption (p = 0.016), smoking (p < 0.001), pica (p < 0.001), paleness (p < 0.001), flattening and pallor of the papillae (p = 0.006), flattening of nail (p = 0.010), and koilonychia (p = 0.006). The rate of regular breakfast intake was higher in male nursing students (55.8%) compared to female nursing students (35.7%) and was statistically significant compared to female nursing students (p=0.046). The present study also showed that the proportion of male nursing students who regularly consume red meat, fish, and chicken was higher than female nursing students (male: 54.9%; female: 41.1%). Table 1 presents the demographic data and clinical characteristics of the groups. The mean hemoglobin level was 15.85 ± 1.10 g/dL in the male student group and 12.90 ± 1.05 g/dL in the female student group. The mean MCV and vitamin B12 levels of the male student group were 85.71 ± 5.99 and 356.19 ± 111.07 pg/mL, respectively. The mean MCV and vitamin B12 levels of the female student group were determined as 84.25 ± 5.32 and 381.41 ± 106.70 pg/mL, respectively. The mean serum iron values of the male and female student groups were 107.60 ± 41.47 μg/dL and 77.38 ± 32.05 μg/dL, respectively. The mean ferritin value of the male student group was 56.4 (36.1) ng/mL, and that of the female student group was 16.3 (14.4) ng/ml. There was a statistically significant difference between male nursing students and female nursing students with respect to hemoglobin (p < 0.001), serum iron (p < 0.001), UIBC (p < 0.001), ferritin (p < 0.001), and transferrin saturation (p < 0.001) (Table 2). Of the female nursing students, 43 (76.8%) had regular menstrual periods, while 13 (23.2%) had irregular menstrual periods. Four (7.1%) of the female nurses had breakthrough bleeding, three (5.4%) were using contraceptives, and none were pregnant.

**Table 1:** Comparison of laboratory parameters and diagnoses between the male and female students Data are presented as mean ± standard deviation, median (interquartile range) or n (%) IDA: Iron deficiency anemia; MCV: mean corpuscular volume; UIBC: unsaturated iron-binding capacity

Parameters	Male students (n = 43)	Female students (n = 56)	p-value
Hemoglobin, (g/dL)	15.85±1.10	12.90±1.05	<0.001
MCV, (fl)	85.71±5.99	84.25±5.32	0.205
Serum iron, (µg/dL)	107.60±41.47	77.38±32.05	<0.001
UIBC, (µg/dL)	232.35±61.08	302.86±52.41	<0.001
Ferritin, (ng/mL)	56.4 (36.1)	16.3 (14.4)	<0.001
Vitamin B12, (pg/mL)	356.19±111.07	381.41±106.70	0.255
Folic acid, (ng/mL)	9.71 (6.24)	10.86 (6.19)	0.092
Transferrin saturation, (%)	42.93 (37.6)	25.83 (20.6)	<0.001
Parasite in stool	0	0	N/A
Occult blood in stool	2 (4.6%)	2 (3.5%)	0.586
Anemia other than IDA	2 (4.6%)	2 (3.5%)	0.586
IDA	0 (0%)	4 (7.1%)	0.030
Iron deficiency	0 (0%)	22 (39.2%)	<0.001

**Table 2:** Clinical findings and anamnesis data of the male and female nursing students Data are presented as mean ± standard deviation or n (%)

Clinical and anamnesis' data	Male students (n = 43)	Female students (n = 56)	p-value
Age, (years)	17.4±0.58	17.64±0.73	0.070
Height (cm)	176.65±7.13	165.16±5.22	<0.001
Weight, (kg)	68.12±11.62	56.73±8.38	<0.001
Accommodation			
With family	39 (90.6%)	48 (85.7%)	0.451
Student dormitory	4 (9.3%)	8 (14.2%)	
Regular breakfast intake			
Yes	24 (55.8%)	20 (35.7%)	0.046
No	19 (44.2%)	36 (64.3%)	
Eating red meat, fish, and chicken regularly			
Yes	28 (54.9%)	23 (41.1%)	0.018
No	15 (34.9%)	33 (58.9%)	
Daily tea consumption			
1-3 glasses	18 (41.8%)	37 (66.0%)	0.016
3-5 glasses	12 (27.9%)	15 (26.7%)	
>6 glasses	13 (30.2%)	4 (7.1%)	
Coffee consumption	31 (72.0%)	37 (66.0%)	0.522
Smoking	2 (4.6%)	22 (39.2%)	<0.001
Pica	18 (41.8%)	37 (66.70%)	<0.001
Examination findings			
Paleness	2 (4.6%)	23 (41.0%)	<0.001
Tachycardia	3 (6.9%)	5 (8.9%)	0.724
Flattening and pallor of the papillae	0 (0%)	9 (16.0%)	0.006
Flattening of nail	0 (0%)	8 (14.2%)	0.010
Koilonychia	0 (0%)	9 (16.0%)	0.006
None	40 (93.0%)	29 (51.7%)	<0.001

ID was detected in 22 (22.2%) female nursing students, and anemia was detected in four (4%) nursing students. While IDA was detected in four (4%) of the female nursing students, IDA and ID were not detected in the male nursing students. Students were divided into four groups according to their diagnoses (normal, ID, IDA, and other anemia) (Table 3). There were statistically significant differences in hemogram (p < 0.001), MCV (p < 0.001), serum iron (p = 0.001), UIBC (p < 0.001), ferritin (p < 0.001), vitamin B12 (p = 0.005), folic acid (p = 0.047), and transferrin saturation values (p < 0.001). Hemoglobin values in the normal, ID, IDA, and other anemia groups were 14.99 ± 1.51 g/dL, 13.01 ± 0.66 g/dL, 11.23 ± 0.52 g/dL, 11.88 ± 1.09 g/dL, respectively. We found that the hemoglobin level in the IDA group was statistically significantly lower than the ID group (p < 0.001). In addition, MCV, vitamin B12, folic acid, serum iron, and ferritin were reduced in the IDA population compared with the ID group, whereas UIBC was significantly higher in the IDA group (357.00 ± 31.19 µg/dL vs. 335.64 ± 35.14 µg/dL; p < 0.001).

**Table 3:** Comparison of students’ laboratory findings between iron deficiency, iron deficiency anemia, other anemia, and normal groups Data are presented as mean ± standard deviation, median (interquartile range) or n (%) MCV: Mean corpuscular volume; UIBC: unsaturated iron-binding capacity, IDA: iron deficiency anemia; ID: iron deficiency

Parameters	Normal (n = 69)	ID (n = 22)	IDA (n = 4)	Other anemia (n = 4)	p-value
Hemoglobin, (g/dL)	14.99±1.51	13.01±0.66	11.23±0.52	11.88±1.09	<0.001
MCV, (fl)	86.32±3.44	84.73±2.92	75.83±11.08	69.95±11.51	<0.001
Serum iron, (µg/dL)	97.71±37.25	66.27±30.93	64.75±23.43	125.25±60.31	0.001
UIBC, (µg/dL)	248.84±55.58	335.64±35.14	357.00±31.19	242.25±102.13	<0.001
Ferritin, (ng/mL)	44.8 (39.9)	10.2 (4.8)	6.7 (4.6)	80.2 (103.7)	<0.001
Vitamin B12, (pg/mL)	359.03±103.3	383.91±77.64	317.75±29.48	546.25±228.917	0.005
Folic acid, (ng/mL)	10.38 (5.57)	10.45 (3.43)	9.70 (4.46)	17.16 (6.23)	0.047
Transferrin saturation, (%)	36.45 (22)	17.22 (16.7)	18.38 (13.3)	69.04 (82.7)	<0.001

## Discussion

An estimated 1.2 billion individuals across the world are affected by IDA, which is considered a treatable public health problem rather than a disease [11]. In regions with low and medium socioeconomic conditions, the primary cause of anemia remains inadequate and unbalanced nutrition. In our study, the frequency values of IDA and ID were found to be 4.0% and 22.2%, respectively. We determined that there were more nursing students with ID than those with IDA. Uncontrolled anemia can lead to impaired cognitive abilities in children, decreased productivity, and potential health problems in adults. Therefore, we deemed it necessary to evaluate our study population in terms of anemia risk.

In IDA, clinical findings secondary to anemia may be found as in all anemias or the diagnosis can be made during laboratory investigations in the absence of any clinical finding. In our study, we found that physical symptoms such as paleness, flattening and pallor of papillae, flattening of nails, and koilonychia were more common in female nursing students. The prevalence of IDA may vary by gender and age. In a study conducted with 1,120 children aged 12 to 16 years, the authors found the prevalence of anemia to be 5.6%. They determined that 59% of anemic patients had IDA, while 41% had a combination of ID and vitamin B12 deficiency anemia [12]. In another study evaluating 1,644 students between the ages of 14 and 21 years, anemia and ID were detected in 11.5% and 18.3% of the girls, respectively, and 1.4% and 1.5% of the boys, respectively, indicating a 10 times higher frequency in the former than in the latter [13]. In the current study, we found that ID was more common than IDA. Furthermore, we observed that the serum iron level of the male nursing student group was higher than that of the female nursing student group. The age group in our study represents individuals who are susceptible to anemia due to factors that facilitate the development of anemia, such as unbalanced and irregular nutrition, unhealthy diets, and the onset of menarche. Nurses play a crucial role in providing frontline care. Therefore, it is of utmost importance to educate nursing students about adequate nutrition and balanced nutrition.

Healthy food that contains heme and non-heme iron such as fat, meat, proteins, bread, fiber, grains, pulses, legumes, fruits, vegetables, and vitamins is necessary to provide energy and enhanced iron absorption. In our current study, regular breakfast intake and regular consumption of red meat, fish, and chicken were found to be statistically significant in male nursing students compared to female nursing students. Our findings showed that the prevalence of IDA and ID was higher among female nursing students. Possible causes of the high prevalence rate of IDA and ID among the female population may include low intake of iron-rich food, poor bioavailability, menarche, dislike of served food, cutting calories to lose weight, lack of awareness of iron deficiency, and nutritional status. Inappropriate dietary choices and frequent consumption of tea, coffee, and cola with meals are associated risk factors for anemia [14]. In our study, tea and coffee consumption was higher among female nursing students than male nursing students. Similar to previous studies that reported that tea intake was significantly higher among anemic subjects [15], we found that there was a statistically significant difference in tea consumption in female nursing students with ID and IDA in our study. In another study with similar results to our study, no significant relationship was found between coffee consumption and anemia risk [16]. Smoking habit is associated with a higher prevalence of IDA in many studies [15]. Cigarette smoking causes an increase in hemoglobin levels, probably mediated by exposure to carbon monoxide which reduces oxygen tension and causes hypoxia in the body [17]. Hypoxia consequently increases the production of erythrocytes from blood-forming organs and elevates levels of hemoglobin, while serum ferritin may be low [17]. In this study, we found that the smoking rate was higher in female nursing students.

In a study investigating the frequency of ID and IDA among nursing students, 112 female students aged 18-25 years were divided into three groups according to their iron and ferritin levels: normal, ID, and IDA. ID was detected in 34% of the students, and IDA in 11%. A high level of statistically significant difference was detected between the ferritin, serum iron, and UIBC values of these three groups. The mean hemoglobin value of the students was 12.26 ± 1.34 g/dL, and their mean ferritin value was 24.55 ± 17.23 ng/mL [18]. In another study, Shams et al. examined the blood parameters of 295 female medical students between the ages of 18 and 25 years. They reported the mean hemoglobin value as 13.7 ± 1 g/dL, MCV as 84.9 ± 3.48 fl, ferritin as 27.7 ± 25.6 ng/mL, iron as 83 ± 41 µg/dL, UIBC as 437 ± 70 µg/dL, and transferrin saturation as 19.8 ± 10%. The authors divided the students into three groups (normal, ID, and IDA) and found statistically significant differences between their ferritin, iron, and transferrin saturation levels (p < 0.05) [19]. In the current study, the nursing students were divided into four groups according to their findings (normal, ID, IDA, and other anemia). Similar to the previous study, we found statistically significant differences in the ferritin, iron, and transferrin saturation levels of these groups (p < 0.05). ID usually develops slowly and insidiously. Many patients have no specific complaints. While IDA can be detected only by measuring hemoglobin levels, ID is often overlooked, despite its higher prevalence. Therefore, it is extremely important to educate nursing students and raise their awareness about ID and IDA.

Our study has certain limitations, the most important of which is the limited size of the population in terms of the number of participants. The main reasons for this limitation were the single-center nature of our study and the sample consisting of only nursing students working in the internal medicine clinic.

## Conclusions

IDA is a public health problem that can be effectively addressed through the implementation of healthy and balanced diets and/or replacement therapies in individuals without any additional pathologies. Our study revealed a higher prevalence of nursing students with ID compared to those with IDA. Considering the sociodemographic characteristics of our study population, they constitute a group that should be given special attention as individuals who will affect public health in relation to their chosen profession. Given the effects of subclinical anemia, there is a need for further research involving nursing students.
